# The Fastest and Most Reliable Identification of True Hybrids in the Genus *Pisum* L.

**DOI:** 10.3390/life13112222

**Published:** 2023-11-18

**Authors:** Hatice Sari, Tuba Eker, Duygu Sari, Munevver Aksoy, Melike Bakır, Veysel Dogdu, Cengiz Toker, Huseyin Canci

**Affiliations:** 1 Department of Field Crops, Faculty of Agriculture, Akdeniz University, Antalya 07070, Turkey; 2 Department of Crop and Soil Science, Washington State University, Pullman, WA 99164, USA; 3 Department of Agricultural Biotechnology, Faculty of Agriculture, Akdeniz University, Antalya 07070, Turkey; 4 Department of Agricultural Biotechnology, Seyrani Faculty of Agriculture, Erciyes University, Kayseri 38039, Turkey

**Keywords:** *xenia*, interspecific crosses, *Pisum sativum*, *P. elatius*, *P. fulvum*

## Abstract

After crosses, the identification of true hybrids is not only the most important step in the initiation of a breeding program but also plays a crucial role in the improvement of hybrid varieties. However, current morphological or molecular-based hybrid identification methods are time-consuming and costly approaches that require knowledge and skill, as well as specific lab equipment. In the current study, *xenia*, direct or immediate effect of pollen on seeds was used to identify true hybrids in the genus *Pisum* L. for the first time without growing F_1_ plants. The current study was therefore aimed to (i) elucidate the *xenia* effect on seeds in intra- and interspecific crosses between *P. sativum* L. subsp. *sativum* var. *sativum* or var. *arvense* L. Poir. and its wild relatives, including *P. sativum* subsp. *elatius* (M. Bieb.) Aschers & Graebn. and *P. fulvum* Sibth. & Sm., and (ii) illuminate the beneficialness of the *xenia* effect in a practical improvement of the genus *Pisum* L. The pea cultivars, including *P. sativum* subsp. *sativum* var. *sativum* and *P. sativum* subsp. *sativum* var. *arvense,* were therefore crossed with *P. sativum* subsp. *elatius* and *P. fulvum*, and the occurrence of the *xenia* effect was studied on the seeds of fertilized female plants immediately after the crosses. It was concluded that using the *xenia* effect for the early detection of true hybrid immediately after crossing was not only the fastest, most reliable, and least expensive option as early selection criteria, but that *xenia* also provided information about dominant seed and pod traits after double fertilization.

## 1. Introduction

The genus *Pisum* L. consists of three cultivated pea species or subspecies and varieties such as *P. sativum* L. (garden pea), *P. arvense* (L.) Poiret. (field pea), and *P. abyssinicum* (A. Braun.) Govorov (dekoko or Ethiopian pea) and two wild species such as *P. elatius* M. Bieb. and *P. fulvum* Sibth & Sm. It was taxonomically classified as *P. sativum* L. subsp. *sativum* (including var. *sativum* and var. *arvense*), *P. sativum* subsp. *abyssinicum* A. Br., *P. sativum* subsp. *elatius* (M. Bieb.) Aschers. & Graebn. (including var. *elatius*, var. *brevipedunculatum* and var. *pumilio*) and *P. fulvum* [1,2]. *Pisum sativum*, the cultivated pea, including garden and field peas, ranks first among cool season food legumes based on production quantity, with 14.6 million metric tons as dry pea and 19.9 million metric tons as vegetables in 2021 [3]. It is cultivated for its dry seeds for human and animal consumption due to high levels of protein and vitamin content in its seeds and pods. It is also cultivated for its green pods and seeds as a vegetable, such as snap beans, in countries of the Mediterranean basin [4]. When used as a rotation crop in small grain cereal based cropping systems, it also reduces diseases, weeds, and the usage of chemical fertilizers owing to its ability to fix atmospheric nitrogen into the soil for the following crop season [5].

According to the available literature, the first record on hybridization in pea was published by A. Knight in 1799, and later studies were chronologically narrated by Smykal [6]. Genetics of seven traits or “elements” was studied by Gregor Johann Mendel in intraspecific crosses in the cultivated pea, and the results of his studies were published in 1866 under the original title “Versuche über Pflanzen-Hybriden” (“Experiments in Plant Hybrids”) [7,8,9,10]. *Xenia*, direct or immediate effects of the fertilizing pollen on endosperm and embryo (inside of embryo sac) of the female plant, was coined as early as 1881 by Wilhelm Olbers Focke [11]. It plays important roles in plant breeding related to double fertilization (embryo and endosperm development), because half of the genome of the embryo and one third of the genome of the endosperm come from the pollen. *Metaxenia* was called the pollen effect on mother tissue (outside of embryo sac) after double fertilization. After a double-fertilization event, the fertilized egg cell becomes the embryo, and the fertilized central cell develops into the endosperm. Fertilization also triggers the development of maternal integument in the seed coat [12]. Although the molecular mechanism of the xenia effect is not clearly defined, there are hypotheses on the involvement of RNAs (small RNA or mRNA). According to these hypotheses, during fertilization, pollen may release miRNAs and/or mRNAs, and these RNAs may diffuse into the mother tissues. These RNAs may involve regulation of gene expression in the mother tissues; translocated RNAs may cause changes in size, shape, color, developmental timing, and chemical composition of seeds and fruits, varying according to the particular male parent [13]. Jafari et al. show that miRNAs are involved in seed size development in almond [14]. Therefore, it is plausible to suggest that RNAs released by pollen may diffuse into the mother tissue and be involved in seed development in peas as well.

Despite various definitions of *xenia* and *metaxenia*, the term *xenia* consists of *metaxenia*. The differences in size, shape, color, developmental timing, and chemical composition of seeds and fruits found as a result of fertilization by different pollens are called the *xenia* effect [15]. *Xenia* refers to the direct or immediate effect of pollen on seeds and fruits that occurs outside of the embryo, while the effect of pollen on the testa and fruit wall is known as *metaxenia* [16]. Although the *xenia* phenomenon was reported in intraspecific crosses among cultivated peas by Carl Correns in 1899, neither *xenia* nor *metaxenia* have been reported in interspecific crosses between cultivated peas and their wild relatives.

Positive effects created by *xenia* or *metaxenia* on yield improvement have been reported in economically important plant species from the 1920s [17] to the new millennium [16,18]. All breeding programs based on crosses have started with intraspecific or interspecific crosses, such as those of the genus *Pisum* [2]. Interspecific crosses are laborious due to the time-consuming emasculation process of the flowers. After crosses, true hybrid plants in F_1_ played crucial roles in the progress of breeding programs. At the initial stages of the seedlings, true hybrid plants in F_1_ must be confirmed to gain time and reduce expenses for the advanced generations. Breeders and researchers rely on adequate dominant morphological traits to distinguish the true hybrid plants among the F_1_ progeny. Differentiation of true hybrid and non-hybrid F_1_ plants based on morphological traits may be impossible due to the similar morphological traits, especially in interspecific crosses, e.g., most of the morphological traits of the wild tall pea (*P. elatius*) are similar to field pea, *P. arvense* [1]. If there are no adequate dominant morphological traits among parents, the use of molecular markers is a fast and reliable option in food legumes [19,20,21] for the elimination of non-hybrid F_1_ plants [21]. Using molecular markers to distinguish the true hybrid plants among the F_1_ progeny requires knowledge, specific lab equipment for molecular techniques, time, and low costs per sample. Despite the low cost per sample, expenses for using molecular markers can run into thousands of dollars due to the high number of samples. However, the developing seed or fruit on a fertilized plant can be examined to determine if it is a true hybrid or not without progressing to F_1_ by determining if there is any *xenia* effect. Immediately after crossing, if the seeds are completely different from the seeds of the selfed mother plant in terms of traits such as shape, color, and size, they could be considered hybrid seeds. When the hybrid seed is determined to be completely different from the mother plant in terms of these traits, there may be no need to grow F_1_ plants and re-test the true hybrid by molecular techniques. Breeders need the fastest, most reliable, and cheapest option as early selection criteria without growing F_1_ plants. Therefore, in the current study, we aimed (i) to show *xenia* on seeds in inter- and intraspecific crosses between *P. sativum* subsp. *sativum* var. *sativum* or var. *arvense* and its wild relatives, including *P. sativum* subsp. *elatius* and *P. fulvum*, and (ii) to emphasize the practical effectiveness of the *xenia* effect in pea breeding.

## 2. Materials and Methods

### 2.1. Parents and Crosses

Despite small differences about the number of subspecies and varieties in the classification [1,2,4,22,23,24,25,26], classification of Smykal et al. [1] and Warkentin et al. [2] was followed in the current study. Five genotypes of cultivated pea, including *P. sativum* subsp. *sativum* var. *sativum* (ACP 11, ACP 20) and *P. sativum* subsp. *sativum* var. *arvense* (ACP 01, ACP 13, ACP 101) were used as female or male parents in intraspecific crosses, while four species of wild peas, consisting of two accessions of *P. sativum* subsp. *elatius* (AWP 440, AWP 442) and two accessions of *P. fulvum* (AWP 600, AWP 601) were pollen donor in interspecific crosses. Twenty plants from each parent genotype were used for crosses. Crosses combinations shown in Table 1 were performed to investigate *xenia* effect.

Before crosses between the cultivated species and the wild species, the following studies [27,28,29,30,31] were taken into consideration due to successful interspecific crosses, especially between the cultivated pea and *P. fulvum*. That is, during crosses, the flowers of the female plants were in the early bud stage, but the flowers of the pollen donor parents were at the end of the bud stage. Corolla in female parents was almost equal to the calyx tube. In the morning, the anthers were gently removed without damaging the sepals and petals. After all emasculations, pollen grains were contacted by the stigma of the female plants within an hour in 2016. Soon after crossings, tags consisting of the names of the parents and the date of crossing were hung on the peduncle of the female plants. Neither plant growth regulators nor additional chemicals were applied to the crossed flowers, but the plants were routinely irrigated to remove drought stress in the glasshouse. Each individual seed of female plants was evaluated in terms of shape, color, width, and length to determine if there was any *xenia* effect.

**Figure 1:**
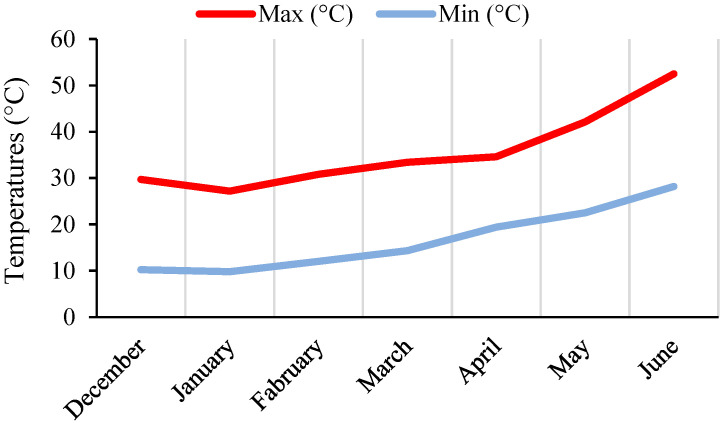
Monthly minimum (Min) and maximum (Max) temperatures in the glasshouse in 2016–2017.

In intraspecific crosses, four F_1_ plants were created in ACP 20 × ACP 101, three in ACP 20 × ACP 01, and two in ACP 11 × ACP 101. In interspecific crosses, two, six, four, and five F_1_ plants were created in ACP 13 × AWP 600, ACP 20 × AWP 600, AWP 442 × AWP 601, and ACP 101 × AWP 440, respectively. F_1_ plants were grown under glasshouse conditions. The plants were irrigated regularly with a drip irrigation system to avoid drought. Weeds were removed manually. The minimum and maximum temperatures for the months in which the F_1_ plants were grown are presented in Figure 1.

### 2.2. Xenia Effects

In the current study, the term *xenia* was considered the immediate effect of the male plant (effect of pollen) on all maternal tissues, including the surface of fruits and seeds after fertilization [15,16]. After crosses, mature pods on female plants were harvested as a single pod to get rid of non-hybrids, and F_1_ plants were grown for checking true hybrids under glasshouse conditions. Each progeny derived from inter- and intraspecific crosses was grown in rows as F_1_ plants in 2017 and tested to determine whether these were true inter- and intraspecific hybrids or not, using morphological traits and molecular data as well.

As quantitative traits, the length and width of each hybrid seed and its parents were recorded (in mm) with a digital caliper. As qualitative traits, the seed color, and seed surface (smooth or wrinkled) were also recorded.

### 2.3. SSR-Based Genotyping for Hybrid Confirmation

DNA isolation was carried out according to the protocol described by Lefort et al. [32] using young, fresh leaves of all hybrids in F_1_ and parents. NanoDrop^®^ ND-1000 Spectrophotometer was used to evaluate the quality and quantity of the isolated DNA (NanoDrop Technologies, Wilmington, DE, USA). Seven SSR markers (D21, AA205, AA355, AD61, AC58, AD59, AD146) developed by Loridon et al. [33] were used to test the true hybrids. The sequences of the polymorphic SSR primers are given in Table 2. Allele size of SSR markers were determined in all hybrids in F_1_s and parents via PCR reaction with M13-tailed primer. A tail (M13 universal sequence (−21), TGT AAA ACG ACG GCC AGT) was added to 5′ end of each forward primer. PCR amplification (15 μL) was conducted using following components: 90 ng genomic DNA; 5 μM of each SSR primer; 5 μM labeled M13 (−21) universal primer; 2.5 mM of each dNTPs; 5X DreamTaq Green Buffer (2 mM MgCl_2_); 5U DreamTaq DNA Polymerase (Thermo Scientific, Waltham, MA, USA). Amplification process included the following steps: 3 min, 94 °C; 35 cycles, 1 min, 94 °C; 1 min, 50–66 °C; 2 min, 72 °C; 8 cycles, 1 min, 94 °C; 1 min, 53 °C; 2 min, 72 °C; 10 min, 72 °C. The M13 (−21) universal primer was 5′-fluorescently tagged with HEX, 6-FAM or ROX. A set of three markers’ PCR products (0.5 μL each) were mixed with 0.5 μL GeneScan-600 LIZ size standards and 9.5 μL Hi-Di™ formamide; denatured at 95 °C for 5 min; and run on the Applied Biosystems Prism 3500 Genetic Analyzer System.

### 2.4. Statistical Analyses

The data obtained from quantitative traits were analyzed for descriptive statistics and analysis of variance (ANOVA) using SPSS 26 software (SPSS 2016). For each quantitative trait, significant differences between hybrid seeds and seeds of their parents were determined using the Tukey’s test with a probability value of *p*  ≤  0.05. To confirm that the hybrids were true hybrids or non-hybrids, allele size was determined with the aid of GENEMAPPER software v5.0.

## 3. Results

### 3.1. Xenia in Intraspecific Crosses

**Figure 2:**
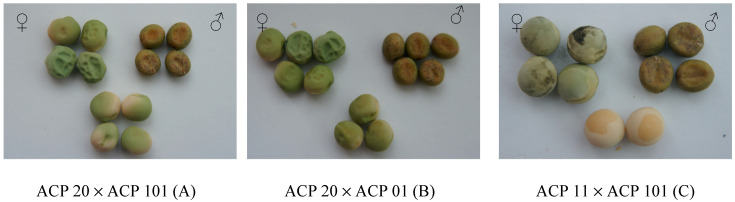
Seed shape and color of parents and hybrids derived from intraspecific crosses between *P. sativum* subsp. *sativum* var. *sativum* × *P. sativum* subsp. *sativum* var. *arvense* (**A**), *P. sativum* subsp. *sativum* var. *sativum* × *P. sativum* subsp. *sativum* var. *arvense* (**B**), and *P. sativum* subsp. *sativum* var. *sativum* × *P. sativum* subsp. *sativum* var. *arvense* (**C**). (♀ is female donor, ♂ is pollen donor).

A total of three intraspecific hybrids were produced between *P. sativum* subsp. *sativum* var. *sativum* (ACP 20) × *P. sativum* subsp. *sativum* var. *arvense* (ACP 101), *P. sativum* subsp. *sativum* var. *sativum* (ACP 20) × *P. sativum* subsp. *sativum* var. *arvense* (ACP 01), *P. sativum* subsp. *sativum* var. *sativum* (ACP 11) × *P. sativum* subsp. *sativum* var. *arvense* (ACP 101) (Figure 2). In intraspecific crosses, four hybrid seeds were obtained from ACP 20 × ACP 101, three hybrid seeds from ACP 20 × ACP 01, and two hybrid seeds from ACP 11 × ACP 101. The accession ACP 20 of *P. sativum* subsp. *sativum* var. *sativum* had a green color and a wrinkled seed coat, and the accession ACP 101 of *P. sativum* subsp. *Sativum* var. *arvense* had a dun color and a smooth seed coat, while hybrid seeds of these parents had a green color and a smooth seed coat (Figure 2A). When the accession ACP 20 (*P. sativum* subsp. *sativum* var. *sativum*) was crossed with the accession ACP 01 (*P. sativum* subsp. *sativum* var. *arvense*), the seeds of the hybrid had a green color and a smooth seed coat (Figure 2B). ACP 11, an accession of *P. sativum* subsp. *sativum* var. *sativum*, had a light green color and smooth seed coat, whereas the accession ACP 101 of *P. sativum* subsp. *sativum* var. *arvense* had a dun color and a smooth seed coat. Seeds obtained from the ACP 11 × ACP 101 crosses had a yellow color and a smooth seed coat (Figure 2C). As a quantitative trait, seed size, including seed width and length, was usually found to differ between values belonging to male and female plants since these traits were not only affected by the male parent but also the female parent. In summary, hybrid seeds obtained from all cross combinations were phenotypically determined to be different from the parent plants and were confirmed as true hybrids.

### 3.2. Xenia in Interspecific Crosses

**Figure 3:**
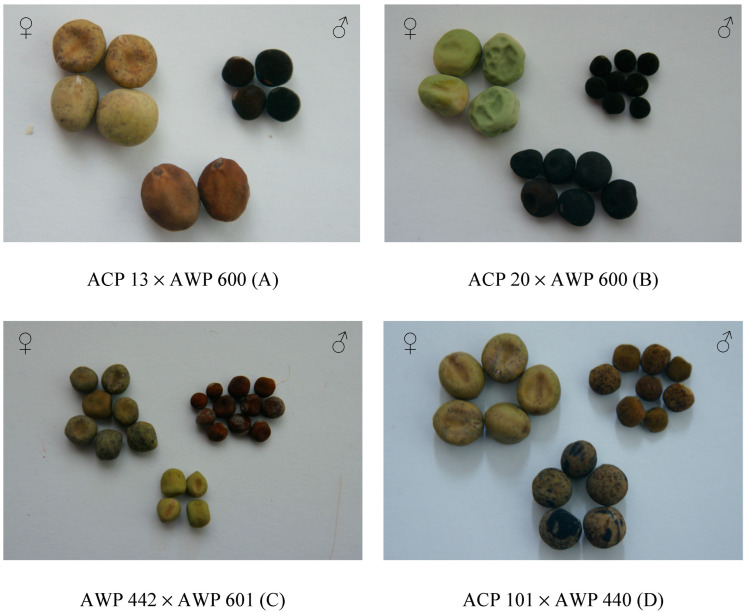
Seed shape and color of parents and hybrids derived from interspecific crosses between *P. sativum* subsp. *sativum* var. *arvense* × *P. fulvum* (**A**)*, P. sativum* subsp. *sativum* var. *sativum* × *P. fulvum* (**B**)*, P. sativum* subsp. *elatius* × *P. fulvum* (**C**)*,* and *P. sativum* subsp. *sativum* var. *arvense* × *P. sativum* subsp. *elatius* (**D**).

Four interspecific hybrids were obtained between *P. sativum* subsp. *sativum* var. *arvense* (ACP 13) × *P. fulvum* (AWP 600), *P. sativum* subsp. *sativum* var. *sativum* (ACP 20) × *P. fulvum* (AWP 600), *P. sativum* subsp. *elatius* (AWP 440) × *P. fulvum* (AWP 601), and *P. sativum* subsp. *sativum* var. *arvense* (ACP 101) × *P. sativum* subsp. *elatius* (AWP 440). In interspecific crosses, two, six, four, and five hybrid seeds were obtained from ACP 13 × AWP 600, ACP 20 × AWP 600, AWP 442 × AWP 601, and ACP 101 × AWP 440, respectively. A wide variation in seed shape and color was observed in interspecific hybrids (Figure 3) when compared to intraspecific ones (Figure 2). The accession (ACP 13) of *P. sativum* subsp. *sativum* var. *arvense* had a smooth seed coat and a dun color, whereas the accession (AWP 600) of *P. fulvum* had a smooth seed coat and a black seed coat (Figure 3). The size of the seeds derived from ACP 13 and AWP 600 was found to be different from both parents. The seeds of this hybrid had a brown color (Figure 3A). The accession ACP 20 of *P. sativum* subsp. *sativum* var. *sativum* had the largest seeds, wrinkled seed coat, and green color, while the accession AWP 600 of *P. fulvum* had small seeds, a smooth seed coat, and a black seed coat, while the seeds of these parents had a black color and a smooth seed coat (Figure 3B). In Figure 3C, the cross between two wild species [*P. sativum* subsp. *elatius* (AWP 442) × *P. fulvum* (AWP 601)] is shown. The accession AWP 442 had a dark green seed coat color with black dots on *testa* and a smooth seed coat, while the accession AWP 601 had a brown seed color. Hybrid seeds obtained between AWP 442 and AWP 601 had a green color without dots on the seed coat (Figure 3C). The seeds derived from *P. sativum* subsp. *sativum* var. *arvense* (ACP 101) and *P. sativum* subsp. *elatius* (AWP 440) had a dun color with a black spotted seed coat, and the size of the seeds was in between the parents, while AWP 440 had a dun seed coat with black spots (Figure 3D).

In interspecific crosses, there were significant differences for all quantitative traits (*p* < 0.05). The seeds of all hybrids were larger than that of their male parents, while they were smaller than that of their female parents, except for ACP 20 × ACP 101 and ACP 13 × AWP 600 crosses. In summary, as in intraspecific crosses, the seeds obtained in interspecific crosses were also hybrid; their traits were different from both parents.

### 3.3. True Hybrids by SSRs

Seven SSR markers developed by Loridon et al. [33] were used to confirm of the hybrids. Four (D21, AA205, AC58, and AD59) of the seven SSR markers showed polymorphism between parents. Confirmation of intra- and interspecific hybridization was determined via the SSR analyses (Table 3). The obtained allele data were found to be compatible in the hybrids and parents (Table 3).

## 4. Discussion

**Figure 4:**
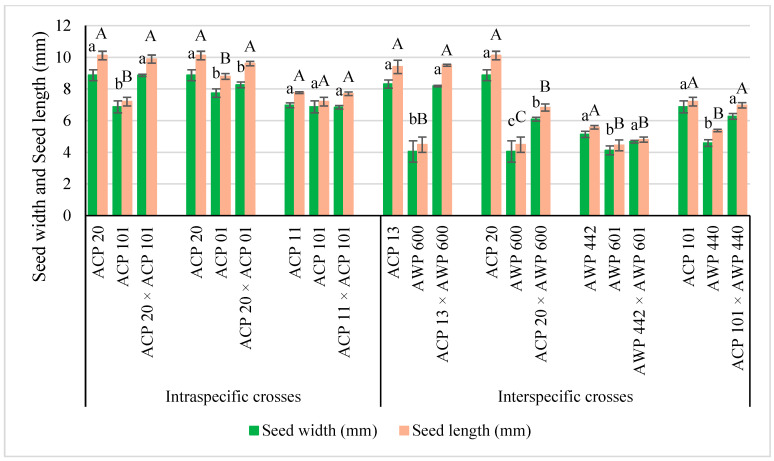
Seed width (mm) and seed length (mm) of parents and hybrids. Lowercase letters indicate compare the hybrids and parents in seed width, whereas uppercase letters indicate compare the hybrids and parents in seed length. Different upper- or lower-case letters are statistically significant (bars show mean ± standard deviations, Tukey’s test; *p* < 0.05).

In the current study, *xenia* effects were determined on seed size, seed color, or seed shape in both intraspecific and interspecific crosses in *Pisum* L. species (Figure 2, Figure 3 and Figure 4 and Table 1). *Xenia* was reported in intraspecific crosses in pea prior to the current study by Carl Correns [34,35], but this is the first report of *xenia* in interspecific crosses. According to the available literature records, *matexenia* was reported in date palm (*Phoenix dactylifera* L.) by Swingle [36], and later in other plants [37,38,39].

*Xenia* is not only a genetic and physiological phenomenon but also causes increases in yield and quality traits in plant species. *Xenia* causes an increase in yield in maize along with cytoplasmic male sterility and is called “Plus-Hybrid” by Weingartner et al. [18,40]. Duc et al. [41] reported that a *xenia* effect in faba bean (*Vicia faba* L.) was observed on the number of cotyledon cells and on seed weight, which was dependent on the male parent. The *xenia* effect was correlated with heterosis in F_1_ plants of faba bean [41]. *Xenia* has been reported in two *Brassica* species [42]. The effect of *xenia* was also studied on 1000-seed weight in rye (*Secale cereale* L.), and it was reported that in xenic hybrids, most of the analyzed traits were insignificantly lower than the standard cultivar [43]. Three lines of registered wheat (*Triticum aestivum* L.) with blue aleurones have been reported to show a strong *xenia* effect ranging from light blue to dark blue when crossed with white and red colored aleurones [44]. Rai et al. [45] studied the *xenia* effect on seed set and grain iron and zinc density in pearl millet (*Pennisetum glaucum* L.). The seed color and smooth surface of the hybrid presented in Figure 2A were considered to be a *xenia* effect, as they were similar to the pollen donor (Figure 2A). Similarly, in the hybrid presented in Figure 2B, it was considered that there was a *xenia* effect in the hybrid since the seed color and smooth surface were similar to the pollen donor (Figure 2B). The *xenia* effect seen on the color of seed in the current study is similar to the findings on seed color in maize (*Zea mays* L.) [18,40,46,47,48,49,50,51,52,53]. The seed coat of the hybrid presented in Figure 2C comes from the pollen donor parent; the hybrid therefore had a *xenia* effect. However, the seed color of the hybrid was different from both its mother plant and the pollen donor (Figure 2C). Similarly, Gartner [54] reported that when a yellow-seeded (female) pea was crossed with a green-seeded pea (male), their offspring had different seed colors from their parents.

Smykal [6] reviewed the crosses in the peas chronologically. A hybrid progeny with dark-colored seeds was obtained as a result of crossing peas with dark grain (gray) into light-colored (white) peas. It is understood that the dark seed color is generally dominant over the light seed color in peas. In this study, dark seed color was seen as a *xenia* effect immediately after crossing. When peas with wrinkled seed shapes were crossed with peas with smooth seed shapes, a hybrid progeny in the form of smooth seeds was obtained [6]. In the current study, similar results were obtained regarding seed shape and color (Figure 2 and Figure 3).

The *xenia* effect is important both in field crops and horticultural crops such as coconut, *Cocos nucifera* L. [55], blueberry, *Vaccinium* species [38], pear, *Pyrus* species [56], and grape, *Vitis vinifera* L. [57]. It was used for practical breeding studies in important crop plants including poppy, *Papaver somniferum* L. [58], *Brassica* species [59], blueberry, *Vaccinium* sp. [60], apple, *Malus domestica* Borkh. [61,62], cucumber, *Cucumis sativus* L. [39], hazelnut, *Corylus avellana* L. [63], date palm [64,65], and mandarin, *Citrus reticulate* Blanco [66]. Piotto et al. [16] described *xenia* and *matexenia* effects on trichome density in tomato fruit and also on seed weight and shape. Six peas were crossed with all combinations, including reciprocals, by Davies [67]. In almond, several miRNAs were identified that are involve in kernel size determination [14]. By performing different crosses between almond cultivars with small and large kernel sizes, Jafari et al. [14] were able to find the target genes of the miRNAs. They concluded that expression of the target genes changed between crosses and developmental stages, suggesting these genes could be involved in kernel size determination, which the researchers called the *xenia* effect. The involvement of miRNAs in seed size determination was also shown in *Arabidopsis* and rice [68,69]. These results suggest that miRNAs could be involved in the *xenia* effect in pea plants as well. Investigation of miRNAs and their target genes could be useful in deciphering the mechanism of the *xenia* effect in pea.

The companies producing commercial hybrid seeds must determine that the seeds are true hybrids before placing them on sale. For this purpose, molecular markers are used to select true hybrid plants in each cross. Expensive infrastructure and laboratories are needed to use molecular markers, and they also need trained technical personnel to interpret results. In addition, it is imperative to determine whether the hybrids performed at the beginning of a hybridization program are true hybrids with the help of morphological or molecular markers to save time and effort. It was proved by this study that the effects of *xenia* will reflect success in hybridization without growing F_1_ plants. In tomato, Piotto et al. [16] proved the *xenia* effect in interspecific crosses. *Xenia* was perceived as a very useful method to detect true hybrids instead of expensive, time consuming and laborious molecular methods.

By 2050, the number of people in the world is expected to reach nine billion [70]. With the increasing population, the need for food will naturally rise. In order to meet the increasing food needs, many methods such as earliness [71], resistance to a/biotic stresses for high yield [72,73,74,75], and speed breeding to obtain more than one generation in a year [76,77] have been developed and used. Six generations of wheat (*Triticum aestivum* and *Triticum durum*), barley (*Hordeum vulgare*), chickpeas (*Cicer arietinum*), peas (*P. sativum*), and four generations of canola (*Brassica napus*) have been obtained in a year via speed breeding [76,77]. In speed breeding, the *xenia* effect can be used to speed up the breeding process by maximizing the genetic diversity of plants in a shorter amount of time. By cross-pollinating different varieties of a plant, breeders can produce offspring with unique genetic combinations that have desirable traits such as higher yields, disease resistance, and improved quality [71,78,79]. This can significantly reduce the time and resources required for traditional plant growth methods and facilitate the development of new crop varieties that can meet the ever-increasing needs for food worldwide.

Early verification of hybrid plants as true hybrids or non-hybrids at the seedling stage in F_1_ is critical for decreasing time and expenses associated with the care of false hybrid plants. In the current study (Table 3), SSRs were used for the determination of hybrids in inter- and intra-specific crosses between cultivated pea and its wild relatives. The maternal parent with cream-colored seeds (ACP 20) and the paternal parent (AWP 600) with black seeds were crossed, and black seeded hybrids were obtained. ACP 20 × AWP 600 hybrid showing the seed color of the paternal parent is the *xenia* effect, and its confirmation of the hybrid was also tested using SSR markers (Figure 3B, Table 3). Black and smooth seed coats were brought about by *xenia* effects, whereas bigger hybrid seeds than those of the male parent (AWP 600) were considered a heterotic effect of the parents (Figure 3B). These results suggest that *xenia* effects can be used to confirm a hybrid when SSR analysis cannot be performed. The initial investigations using SSRs were conducted on common bean (*Phaseolus vulgaris* L.) and soybean (*Glycine max* Merril) utilizing RAPD (Random Amplified Polymorphic DNA) [80]. To detect true F_1_ plants in lentils, Solanki et al. [19] used RAPD and SSR markers (*Lens culinaris* Medik). Kosterin et al. [81] reported that in interspecific crosses using *P. fulvum*, hybrid plants were confirmed using two alleles specific to *P. fulvum* that influence the pod phenotype: the dominant *Astr*, which causes anthocyanin spots on the pod wall, and the recessive *n* gene, which causes a thick, fleshy pod wall in homozygotes. Furthermore, plastid inheritance was evaluated in different hybrids using the presence/absence of the *Asp LEI* (*Hsp AI*) restriction endonuclease recognition site in the *rbcL* gene [81]. Ochatt et al. [82] confirmed the hybrids of interspecific crosses obtained from *P. sativum* × *P. fulvum* by molecular markers. Caballo et al. [21] reliably tested the confirmation of F_1_ hybrids with sequence-tagged microsatellite site (STMS) markers in intraspecific crosses of chickpea (*C. arietinum* L.). Morais et al. [83] utilized SSR to evaluate the efficacy of controlled crossings in common bean. The presence of pigmented flowers, which differed from both parents and were dominant over white-flowered *P. sativum*, was also used to confirm F_1_ plants using morphological markers [84]. Additionally, the kompetitive allele-specific PCR (KASP) markers developed by [85] were safely tested on a validation panel of 39 genotypes of groundnut, which included bold-seeded, small-seeded, and five F_1_ generation lines derived from small- and bold-seeded genotypes. Also, Parmar et al. [86] used the KASP assay to determine for true hybrids of groundnut and confirmed that 66% of the crosses were true hybrid seeds. At the F_1_ stage, molecular markers can be reliably used to confirm that a hybrid is true. However, seeds that are supposed to be hybrids still have to be planted and grown for a period of time. Our results show that immediately after crossing, hybrid seeds are completely different from the seeds of the mother plant in terms of traits such as shape, color, and size, which is an indication of a hybrid. When the hybrid seed is determined to be completely different from the mother plant in terms of these traits, there may be no need to grow F_1_ plants and re-test the hybrid confirmation by molecular techniques. The validation of hybrids with the *xenia* effect is easier, cheaper, and less time-consuming than validation with molecular markers.

## 5. Conclusions

Although the effects of *xenia* have been reported in genetics, physiology, breeding, and agricultural production in the plant species mentioned above, they have not been reported in interspecific crosses among *Pisum* species prior to the current study. According to the outcome of inter- and intraspecific crosses between *Pisum* species, the following conclusions were made: (i) visible morphological differences were detected after crossing that were different from the traits of the parents; (ii) the effects of *xenia* can be used for qualitative and quantitative traits such as seed color and shape, seed width, and length; (iii) the effects of *xenia* on the selection of true hybrids immediately after crosses were not only the fastest, most reliable, and cheapest option as early selection criteria, but *xenia* also reflected some dominant seed and pod traits after double fertilization. True hybrids can be identified immediately after crossing, considering the *xenia* effect.

## Figures and Tables

**Table 1:** Seed color and shape of seed coat of parents and hybrids derived from intra- and interspecific crosses among peas.

Crosses	Species	Parents and Hybrids	Seed Color	Shape of Seed Coat
Intraspecific	*P. sativum* subsp. *sativum* var. *sativum*	ACP 20 (♀)	Green	Wrinkled
	*P. sativum* subsp. *sativum* var. *arvense*	ACP 101 (♂)	Dun	Smooth
		Hybrid	Green	Smooth
	*P. sativum* subsp. *sativum* var. *sativum*	ACP 20 (♀)	Green	Wrinkled
	*P. sativum* subsp. *sativum* var. *arvense*	ACP 01 (♂)	Brown-dun	Smooth
		Hybrid	Green	Smooth
	*P. sativum* subsp. *sativum* var. *sativum*	ACP 11 (♀)	Green	Smooth
	*P. sativum* subsp. *sativum* var. *arvense*	ACP 101 (♂)	Dun	Smooth
		Hybrid	Cream	Smooth
Interspecific	*P. sativum* subsp. *sativum* var. *arvense*	ACP 101 (♀)	Dun	Smooth
	*P. sativum* subsp. *elatius*	AWP 440 (♂)	Dun-black spotted	Smooth
		Hybrid	Dun-black spotted	Smooth
	*P. sativum* subsp. *sativum* var. *arvense*	ACP 13 (♀)	Brown-dun	Smooth
	*P. fulvum*	AWP 600 (♂)	Black	Smooth
		Hybrid	Dark brown	Smooth
	*P. sativum* subsp. *sativum* var. *sativum*	ACP 20 (♀)	Green	Wrinkled
	*P. fulvum*	AWP 600 (♂)	Black	Smooth
		Hybrid	Black	Smooth
	*P. sativum* subsp. *elatius*	AWP 442 (♀)	Dark green-black spotted	Smooth
	*P. fulvum*	AWP 601 (♂)	Brown	Smooth
		Hybrid	Green	Smooth

**Table 2:** The sequences of the polymorphic SSR primers used in the study.

PRIMERS	FORWARD (5′-3′)	REVERSE (3′-5′)
D21	TATTCTCCTCCAAAATTTCCTT	GTCAAAATTAGCCAAATTCCTC
AA205	TACGCAATCATAGAGTTTGGAA	AATCAAGTCAATGAAACAAGCA
AC58	TCCGCAATTTGGTAACACTG	CGTCCATTTCTTTTATGCTGAG
AD59	TTGGAGAATGTCTTCTCTTTAG	GTATATTTTCACTCAGAGGCAC

**Table 3:** SSRs allele data of some inter- and intraspecific crosses among *Pisum* species and their parents.

Crosses	Species	Parents and Hybrids	SSRs			
			D21	AA205	AC58	AD59
Intraspecific	*P. sativum* subsp. *sativum* var. *sativum*	ACP 20 (♀)	218/292	253	219/222	339
	*P. sativum* subsp. *sativum* var. *arvense*	ACP 101 (♂)	296	249	228	339
		Hybrid	218/296	249/253	219/228	339/339
	*P. sativum* subsp. *sativum* var. *sativum*	ACP 20 (♀)	218/292	253	219/222	339
	*P. sativum* subsp. *sativum* var. *arvense*	ACP 01 (♂)	296	253	228	NA
		Hybrid	218/296	253/253	219/228	339
Interspecific	*P. sativum* subsp. *sativum* var. *arvense*	ACP 101 (♀)	296	249	228	339
	*P. sativum* subsp. *elatius*	AWP 440 (♂)	260	258	213	339
		Hybrid	260/296	249/258	213/228	339/339
	*P. sativum* subsp. *sativum* var. *sativum*	ACP 20 (♀)	218/292	253	219/222	339
	*P. fulvum*	AWP 600 (♂)	296	224	213/228	335/339
		Hybrid	218/296	NA/224	222/213	339/339

NA: The PCR result of the given parent is not available.

## Data Availability

All data are available in this article.
